# FLEX-IoT: Secure and Resource-Efficient Network Boot System for Flexible-IoT Platform

**DOI:** 10.3390/s21062060

**Published:** 2021-03-15

**Authors:** Keon-Ho Park, Seong-Jin Kim, Joobeom Yun, Seung-Ho Lim, Ki-Woong Park

**Affiliations:** 1 Department of Computer and Information Security, and Convergence Engineering for Intelligent Drone, Sejong University, Seoul 05006, Korea; imguno0629@naver.com (K.-H.P.); sys.ryan0902@gmail.com (S.-J.K.); jbyun@sejong.ac.kr (J.Y.); 2 Division of Computer Engineering, Hankuk University of Foreign Studies, Yongin 17035, Korea

**Keywords:** flexible IoT, secure TFTP, attacker deception, resource-efficient network boot

## Abstract

In an internet of things (IoT) platform with a copious number of IoT devices and active variation of operational purpose, IoT devices should be able to dynamically change their system images to play various roles. However, the employment of such features in an IoT platform is hindered by several factors. Firstly, the trivial file transfer protocol (TFTP), which is generally used for network boot, has major security vulnerabilities. Secondly, there is an excessive demand for the server during the network boot, since there are numerous IoT devices requesting system images according to the variation of their roles, which exerts a heavy network overhead on the server. To tackle these challenges, we propose a system termed FLEX-IoT. The proposed system maintains a FLEX-IoT orchestrater which uses an IoT platform operation schedule to flexibly operate the IoT devices in the platform. The IoT platform operation schedule contains the schedules of all the IoT devices on the platform, and the FLEX-IoT orchestrater employs this schedule to flexibly change the mode of system image transfer at each moment. FLEX-IoT consists of a secure TFTP service, which is fully compatible with the conventional TFTP, and a resource-efficient file transfer method (adaptive transfer) to streamline the system performance of the server. The proposed secure TFTP service comprises of a file access control and attacker deception technique. The file access control verifies the identity of the legitimate IoT devices based on the hash chain shared between the IoT device and the server. FLEX-IoT provides security to the TFTP for a flexible IoT platform and minimizes the response time for network boot requests based on adaptive transfer. The proposed system was found to significantly increase the attack-resistance of TFTP with little additional overhead. In addition, the simulation results show that the volume of transferred system images on the server decreased by 27% on average, when using the proposed system.

## 1. Introduction

According to Gartner, over 20 billion internet-connected devices are expected to operate in 2020 [1]. More recent research [2] projects that this number will increase to 75.44 billion by 2025. Many companies and countries continue to develop internet of things (IoT) technologies, devices, and service platforms composed of numerous IoT devices such as smart homes, smart buildings, and smart cities [3]. IoT platforms, which are composed of an extremely large number of IoT devices, have been experiencing a paradigm shift towards creating flexible environments where plenty of IoT devices can be dynamically deployed according to the varying situation of the platform. Considering the number of IoT devices is soaring [2], we predict that the total volume of system images that need to be transferred increases accordingly. This is why we think that the efficient use of network resource in an IoT platform is an important issue to deal with. A considerable number of research studies have been performed on reinforcing the dynamicity and interoperability of IoT platforms [4,5]. These studies have suggested various meaningful ideas to efficiently make IoT devices perform new roles or actions beyond those defined during their initial deployment. There also have been many studies on how to use network resource as efficiently as possible in IoT environments [6,7]. To contribute toward strengthening the interoperability and dynamicity of IoT platforms, we suggest a method for transferring system images to IoT devices. Our proposed method tries to use network resource as efficiently as possible. As the number of IoT devices is increasing rapidly [2], the total volume of data that needs to be transferred in an IoT platform will increase accordingly. This is why we believe that the efficient use of network resource in an IoT platform is an important issue that needs to be dealt. Furthermore, security incidents [8,9,10] related to IoT devices have become more frequent. This means that there is an urgent need of greater level of security in IoT platforms.

**Figure 1:**
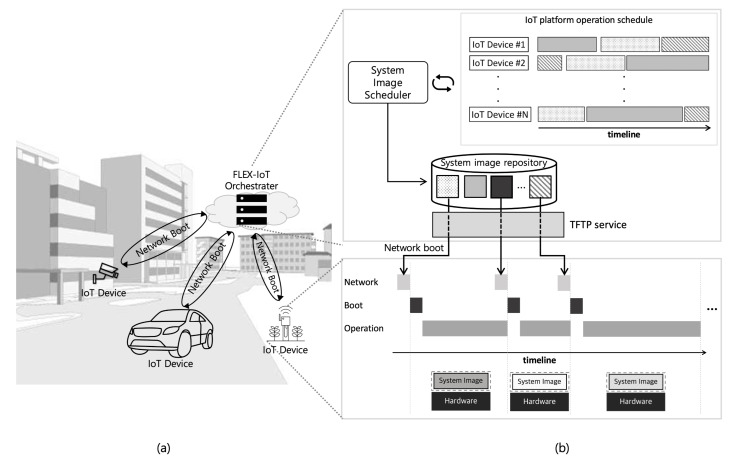
Overview of flexible internet of things (FLEX-IoT) platform. (**a**) Network boot of IoT devices, (**b**) internal process of network boot on FLEX-IoT orchestrater.

Based on the motivations mentioned above, we define a flexible IoT platform, termed FLEX-IoT, and propose a corresponding network boot system. FLEX-IoT platform is shown in Figure 1. Figure 1a shows IoT devices and the FLEX-IoT orchestrater on the FLEX-IoT platform. Figure 1b represents the internal process of managing the FLEX-IoT platform on the server. FLEX-IoT platforms operate based on network boot. The server, termed FLEX-IoT orchestrater, manages the IoT platform schedule of the IoT devices and transmits the planned system image to each IoT device according to the schedule. Each IoT device performs various roles by changing its system image according to its current operational purpose. The system image scheduler establishes an IoT platform operation schedule which includes schedules for all IoT devices on the platform. The FLEX-IoT orchestrater controls each IoT device based on the IoT platform operation schedule, and it allows the FLEX-IoT orchestrater to flexibly operate the IoT devices. This increases device usability, leading to more flexible and dynamic operation of the IoT platform.

However, there are two main obstacles to realizing network boot technology on an IoT platform. Firstly, there are security issues related to trivial file transfer protocol (TFTP). TFTP is typically used for network boot systems such as the pre-boot execution environment [11]. As the TFTP was designed without any explicit security considerations, it has no file access control by default [12]. TFTP is the most commonly used protocol in network boot environments as it provides simple and fast file transfer [13,14]. This makes it difficult to replace the TFTP with other FTPs. Secondly, transmitting a lot of system images to IoT devices in an IoT platform would incur high network traffic consumption. A huge number of IoT devices are hosted on an IoT platform and this number is expected to steadily increase [1,2]. Thus, the need for transmitting system images will increase accordingly, which will, in turn, require more network traffic consumption. This can cause a network transmission delay in an IoT platform, which is fatal for the IoT platforms that require real-time operations. To deal with this problem, there have been a number of research studies focusing on using network resource as efficiently as possible [6,7]. In this regard, we think managing network resource efficiently is an important topic to consider in IoT platforms.

**Figure 2:**
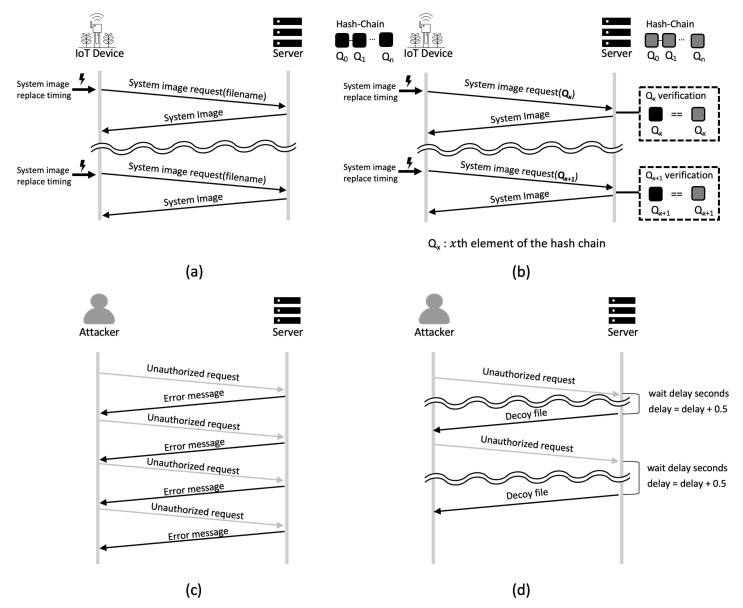
Trivial file transfer protocol (TFTP)-compatible file access control and attacker deception technique. (**a**) System image request and response of native TFTP, (**b**) system image request and response of TFTP-compatible file access control, (**c**) unauthorized file request and error message response of native TFTP, (**d**) unauthorized file request and the attacker deception technique.

**Figure 3:**
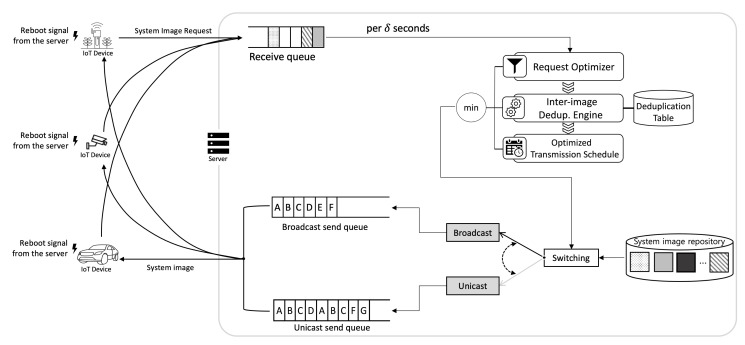
Adaptive transfer.

To overcome these obstacles, we propose a secure and resource-efficient network boot system for FLEX-IoT platform. As shown in Figure 2 and Figure 3, the proposed system improves the security of the TFTP and implements a resource-efficient file transfer technique for system image transfer which is the server’s response to a network boot request message received from the IoT device.

As shown in Figure 2, we design (1) a TFTP file access control function to combat TFTP security threats, (2) an attacker deception technique to provide security against TFTP file acquisition attacks. The proposed TFTP’s file access control is designed to be compatible with the existing TFTP and to be computationally efficient to minimize the overhead induced by file access control when the server receives system image request messages. File access control is designed such that only IoT devices that share a secret value (seed value) with the server and those devices which know the secret value can request a system image through the TFTP. By complying with the design of the existing TFTP, the proposed system does not inhibit the usability of the TFTP. The attacker deception technique increases the time required for an attacker to gain unauthorized files through the TFTP.

Additionally, as shown in Figure 3, we propose an adaptive transfer method to handle demanding network resource consumption in IoT platforms that include a large number of IoT devices. Based on the resource-efficient file transfer method, termed adaptive transfer, we can minimize the network resource consumption for transmitting system images of IoT devices in IoT platforms. This allows the server to transmit the system images by using network resource as efficiently as possible. To this end, the server uses both unicast and broadcast for file transfer. To determine an appropriate transfer method, the server performs request optimization and inter-image deduplication. The request optimizer makes the server transmit the system image by using broadcast when there are heavily duplicated requests for the same system image. The inter-image deduplication engine outputs duplicated system image blocks and extra blocks which can be retrieved from the deduplication table.

Finally, this study is an extension of our previous study [15]. Here, we focus on the implementation and experimentation of the system proposed in the previous study. Our objective in this study, however, was to design a reliable TFTP to improve the security of the FLEX-IoT platform. By implementing the system proposed in the previous study, deduplication table outputs are duplicated, and extra blocks of system images are achieved. Then, the order in which the redundancy of the system images is removed is scheduled so as to minimize the average time required to complete a transmission when a plurality of system images are transmitted. This allows the server to boot IoT devices by responding to all requests within a certain period of time, even if the number of network boot requests increases.

The main contributions of the system proposed in this paper are as follows. Here, the file access control for the system image of the server allows it to be accessed by only legitimate IoT devices. If there are unauthorized system image requests, the server effectively prevents the brute-force attack by using attacker deception technique. Since the proposed system is designed to be compatible with the existing TFTP, it can be immediately applied to the existing IoT platforms. In addition, the proposed system employs network resource as efficiently as possible, which is an important issue to consider to provide interoperability in IoT platforms and for guaranteeing the real-time requirement in a time-sensitive environment.

The remainder of this paper is organized as follows. Section 2 analyzes relevant research work to highlight the limitations of existing systems. Section 3 discusses the design of the proposed system after deriving the requirements for applying network boot to FLEX-IoT platforms. In Section 4, we present the experiments and the performance evaluation of the proposed system. Section 5 presents our conclusions.

## 2. Related Work

### 2.1. Attack-Resistant TFTP

Several studies on improving security for the TFTP in general network environments have been conducted as a result of the recent growth of the IoT industry. Research on TFTP security has mainly focused on encryption. Previous studies have implemented a TFTP option or designed TFTP security with additional encryption functions.

Horvat et al. [14] added new opcode to improve the security of the TFTP in a multi-agent system environment and proposed authentication and data exchange methods based on their opcode. They used challenge-response-type client authentication and proposed authentication and data exchange between the host and the embedded system to improve TFTP security. However, this method has poor compatibility with environments using the existing TFTP as the new opcode for authentication has to be added to the TFTP. In this paper, we propose and implement an access control function for the TFTP compatible with the existing TFTP environments. Isa et al. [13,16] supported various types of cryptographic key exchanges based on a TFTP option extension [17] to improve the security of the TFTP in smart environments, such as IoT environments. New opcode for cryptographic key exchange was added and cryptographic keys are exchanged to encrypt data. Mohamed [18,19] implemented a TFTP based on Diffie–Hellman key exchange and advanced encryption standard public key encryption to improve the security of data and message exchange over the TFTP in machine-to-machine environments. We conducted a study to apply encryption to the data transmitted through this TFTP. A paper on the encryption of data by sharing public-key-based parameters in advance [18] presented preliminary work for exchanging public keys between a client and server, as well as exchanging public keys for client file access requests. In the proposed system, public key exchanges and file access requests are handled separately. In the later work [19], public key exchange was performed as part of the file access request process, which yielded similar performance compared to the previously proposed system. This system provided security against man-in-the-middle attacks by applying encryption to data transmitted via the TFTP, but it did not handle actual file access control because it focused on data exchange security for the TFTP.

Recently, researches on attacker deception techniques have been conducted for active security against cyber attacks. Attacker deception techniques are techniques that deceive an attacker so that they have uncertainty about the attack.

Shu [20] proposed an attacker deception technique to provide security against advanced persistent threat (APT) attacks on FTP services. When an attacker attempts to obtain a file through the FTP, this technique generates a decoy file to respond to the attack. The decoy file was placed according to the attacker’s perceived knowledge. When access to the decoy file occurred, the deception server responded to the attacker’s server to prevent further APT attacks against the FTP. The goals of this method includes designing a decoy file based on an attacker’s perceived knowledge and improving the security of the FTP against deceptive attacks. However, responses through decoy files have limited application since an attacker must access the specific decoy file among many regular files. No system can prepare for all these possibilities. The attacker deception technique proposed in this paper can easily detect attacks based on the TFTP file request message and uses decoy files to prevent attacks.

### 2.2. Resource-Efficient Network Boot

Takada [21] proposed a peer-to-peer (P2P) network boot scheme to eliminate the network bottlenecks induced by network boot in environments where network boot is used to manage many computers such as university classrooms. In this scheme, a client downloads a system image from both a nearby client and the server at network boot time. The client then divides this system image to form a system image block, which is shared through the local area network using a P2P method. After executing this procedure, it performs a network boot. This network boot system improves performance as the number of client nodes increases. However, P2P network boot performance varies depending on the number of participating nodes, which means it does not guarantee consistent performance. Therefore, in our method, to ensure consistent performance regardless of the number of IoT devices participating in network boot on an IoT platform, the server dynamically switches the file transmission method based on the characteristic of system image requests.

Data deduplication technology compresses data by dividing it into several regions, and identifying and removing redundancy between the regions. Deduplication is commonly used to reduce the space required for storing large amounts of data in backup systems or cloud systems [22,23]. It may also be used to reduce network traffic depending on where deduplication is performed within the client-server architecture [24]. Mandagere [25] defined and proposed several items to consider when using deduplication technology. The effectiveness of deduplication technology is heavily dependent on the characteristics of the target platform. Mandagere proposed three considerations for timing, placement, and algorithms. Timing refers to the time at which deduplication is performed, such as the time of day at which deduplication will occur during the data lifecycle. Placement refers to the location at which deduplication is performed within a client-server-store architecture. Algorithms are the mechanisms by which redundancy is removed. Algorithms can be divided into fixed-chunk, variable-chunk, and delta-encoding algorithms. Algorithms should be selected according to the characteristics of the source data. In this study, we aim to minimize the reconfiguration time for booting after file transfer. Therefore, we propose a scheduling method that determines the order of transmission for the system image blocks by considering reconstruction time, which is not a main consideration in the existing data deduplication techniques.

## 3. System Design of FLEX-IoT Platform

**Figure 4:**
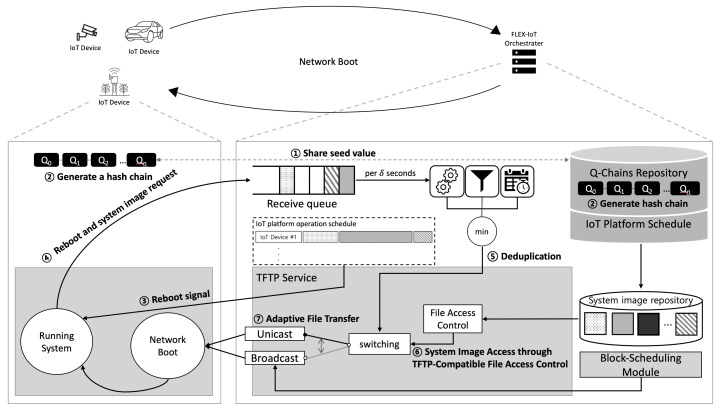
Overall process of the proposed system.

In this section, we present the proposed system for FLEX-IoT platform as shown in Figure 4. Section 3.1 gives an overview of the process of FLEX-IoT platform. In Section 3.2, we introduce TFTP with enhanced security which consists of TFTP file access control and attacker deception technique. Section 3.3 presents resource-efficient network boot which employs adaptive transfer.

### 3.1. The FLEX-IoT Platform Overview

Figure 4 shows the overall process of the proposed system. The FLEX-IoT orchestrater (the server) manages IoT devices based on IoT platform operation schedule. The server maintains the IoT platform operation schedule so that the IoT platform can flexibly deal with various situations. For example, an IoT platform can experience unexpected incidents during operation. In that case, the IoT platform would need to change the operation mode of the IoT devices to appropriately deal with the situation, for which the existing IoT platform operation schedule would need to be modified accordingly. When the system image of an IoT device needs to be changed according to the schedule, the server sends a reboot signal to the IoT device. Then, the IoT device and the server start the network boot process. The details of this boot process is as follows. First, the server and the IoT device share a seed. The seed of the IoT device is contained in the device before it is deployed to the IoT platform. The entities that know the seed value are only the server and the IoT device. This makes the IoT platform more secure as the compromised device, which does not share the seed value with the server, cannot generate the appropriate hash chain. In case of new IoT devices that do not contain the same seed value as the server, which is outside of our security assumption, existing encryption methods may be used instead. The server and the IoT device generate a hash chain by applying multiple hash functions to the seed. The server sends reboot signal to IoT devices according to the IoT platform operation schedule. Then, IoT devices boot the system via network boot. An IoT device sends an element of hash chain to a server through TFTP to request system image. The server verifies the IoT device through the hash value received from the IoT device. This is possible since the IoT device and the server have the same hash chain generated by using the same seed. If the element of the hash chain is legitimate, then the server sends the system image for the IoT device. This is done by setting the name of the system image file for the IoT device on the server as the hash value. The server processes requests on the receive queue per δ seconds and determine the transmission method (unicast or broadcast) based on the degree of duplication among requests and system images.

**Figure 5:**
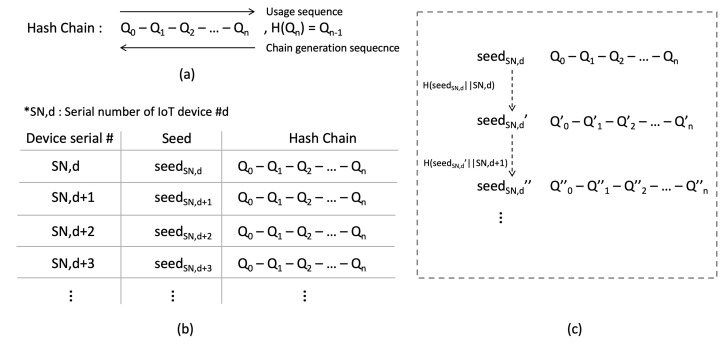
The hash chain. (**a**) Sequence of hash chain generation and usage, (**b**) information on seeds and hash chains of IoT devices stored in the server, (**c**) renewal of hash chain.

Figure 5a shows the sequence of hash chain generation and usage. Each of the IoT device and the server generates a hash chain of length *N* by applying the hash function *N* times to a seed value previously shared between them and the IoT device serial number. As a result that hash chains are created in the reverse order of their use, inverse computation of the hashes is very difficult. Once the hash chain is generated completely, the elements of the hash chains are used one by one to send a system image request and verify the legitimacy of the IoT device. Since there are a large number of IoT devices on the FLEX-IoT platform, servers maintain a data structure which contains seed values, corresponding hash chains of each seed value and device serial numbers, as shown in Figure 5b. In case of using all of N elements of the hash chain, the IoT device and the server can renew its hash chain by generating a new one.

When generating a new hash chain, both parties first generate a new seed value by applying a hash function to the result of concatenating previous seed value and the device serial number ($H(Seed_{SN,d}||SN,d)$). Then, they generate a new hash chain by using a new seed value, as shown in Figure 5c. This enables both the IoT devices and the servers to continue to communicate permanently without having to exchange a new seed value again.

Our system is distinguished by two characteristics. The first is improved TFTP security. We maintain the advantages of the TFTP while supporting secure network boot with minimal security threats. The second is resource-efficient file transfer. Specifically, we propose a resource-efficient file transfer scheme that allows a server to manage network boot requests from a large number of IoT devices within the expected time.

### 3.2. TFTP with Enhanced Security

#### 3.2.1. TFTP-Compatible File Access Control

**Figure 6:**
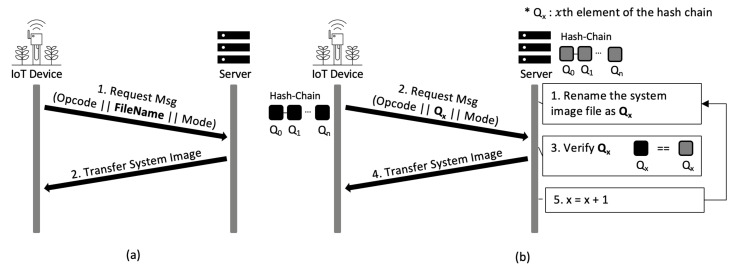
The procedure of TFTP-compatible file access control. (**a**) Flow chart of system image request and response on the native TFTP, (**b**) flow chart of the proposed TFTP-compatible file access control.

There is no mechanism for file access control in the native TFTP. We add file access control to the TFTP so that only the authorized IoT devices can access files. The TFTP supports only minimal features for file transfer. Users cannot see the list of files on the TFTP server since it does not support a command for viewing file lists. Therefore, when requesting a file via the TFTP, a user must know the exact file name on the TFTP. We use this perceived weakness of the system as a security feature. Figure 6 shows the procedure of the TFTP-compatible file access control. The proposed TFTP-compatible file access control has the same protocol as the native TFTP, as shown in Figure 6a. This means that it is fully compatible with the existing TFTP. A hash chain is generated by using a seed value shared between the IoT device and the server. $Q_{x}$ represents the *x*th element of the hash chain. The IoT devices are required to access files on the server by using $Q_{x}$ as the file name. This allows only legitimate IoT devices to access a file via the TFTP since only the server and legitimate IoT devices have hash chain that includes $Q_{x}$ and only the legitimate IoT device and the server know the right element of the chain for the corresponding request message.

Figure 6b shows the flow chart of the TFTP-compatible file access control. The IoT device writes the value of the *x*th element of the hash chain in the file name area of the TFTP read request (RRQ) packet and transmits the packet to the server. The server verifies that the file name matches $Q_{x}$ which is the element of the hash chain supposed to be used for this request message. If the file name and $Q_{x}$ match, the server sends the appropriate system image to the IoT device. Then, the server sets $Q_{x+1}$ to a value to be used at the next request message. This means that the server sets the name of the next system image file of the IoT device to $Q_{x+1}$. At the next request, the server expects the IoT device to use $Q_{x+1}$ as the file name. Since the IoT device and the server change the element of the hash chain they use every time they communicate, each of the elements is used only once.

#### 3.2.2. TFTP-Compatible Attacker Deception

The TFTP-compatible file access control identifies legitimate IoT devices and transfers the necessary files. However, the TFTP does not provide security for file scanning attacks such as brute-force attacks. Therefore, in this paper, we propose an attacker deception technique that makes brute-force attackers spend excessive time on an attack. The proposed attacker deception technique provides security against brute-force attacks that attempt to gain access to files stored on the server through the TFTP. When attackers access a file that does not exist on the server, an error message is returned. The attackers can determine the existence of the file based on the received error messages during the brute-force attack and simply repeat the attack until the file is acquired. The proposed attacker deception technique sends a deception message to an attacker instead of a simple error message if the requested file does not exist. In addition, to increase the time required for the brute-force attack, the server intentionally responds late to the requests considered to be attacks.

**Figure 7:**
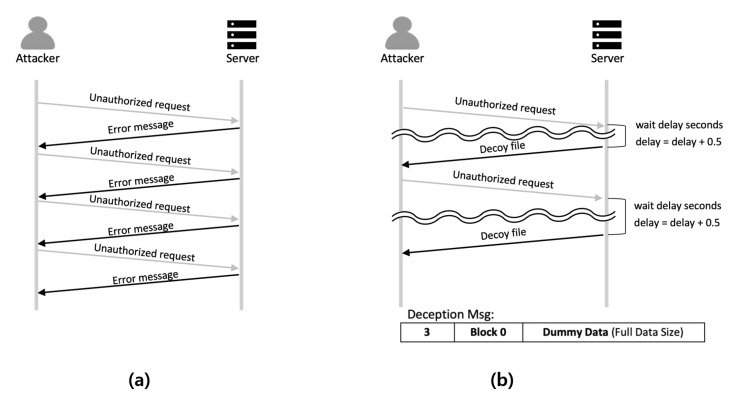
TFTP error handling. (**a**) Native TFTP error handling, (**b**) TFTP-compatible attacker deception technique error handling.

On the TFTP, when a data block smaller than the data block size is transmitted, this block is considered to be the last block of the data. For example, when using 512 bytes as the data block size, the server divides the file to be sent into 512 byte blocks to respond to a client’s file request. When a client receives a block of data smaller than 512 bytes, the client recognizes that the transfer is complete and stops receiving the file as the last part of the file will be smaller than 512 bytes. If the file size is an exact multiple of 512 bytes, the server sends an additional data block of 0 byte to indicate the end of the file. In this situation, when the client receives a data block of 512 bytes, it recognizes that there is an additional data block to be transferred and continues receiving the file. The attacker deception technique proposed in this paper uses this feature of the TFTP. The server sends data blocks with maximum sizes instead of error messages to the attackers to prevent attackers from recognizing that they should repeat other attacks immediately. In addition, the server maintains an integer variable named delay which decides the time the server should wait before transmitting the decoy files. Whenever there is a request message with a file name that does not exist on the server, the value of this variable increases by 0.5 s. The higher the value of delay is, the longer the server waits before it transmits decoy files to the attackers. In addition, the server makes sure that the delay does not become more than 3 s to prevent the time out error. In this way, the server can significantly increase the time required for attackers to perform brute-force attack. Figure 7a shows the flow chart of attacking a server which is using the native TFTP. An attacker can instantly determine whether a file exists on the server by looking at the error message. However, as shown in Figure 7b, when the proposed attacker deception technique is deployed, attackers cannot recognize if the file they received from the server is a decoy file right away. This makes it difficult for the attackers to decide if they should proceed with the next attack. Additionally, as the brute-force attack targeted at the server is repeated, the time taken to receive the response from the server will increase. This leads to an increase in the time interval between attack attempts. Brute-force attack is a basic attack in which a large number of attacks are performed in a short time period. Since the brute-force attackers have to wait between attacks, the proposed method increases the total attack time. This increased attack trial time makes obtaining real files infeasible.

In case an IoT device malfunctions for a time instant, it can be regarded as an attack when the server uses the attacker deception method. However, this does not cause any issue as the IoT device will use the correct element of the hash chain at the next request. In addition, to allow IoT devices to be authenticated again if needed, the server should not ban the devices that request system image with the wrong hash value.

### 3.3. Resource-Efficient Network Boot

#### 3.3.1. Adaptive Transfer

In the FLEX-IoT platform, network boot occurs frequently. When network boot occurs, the server needs resources to respond to every request. The more IoT devices there are in the network, the more resources the server requires. When a large number of network boot requests arrive at the server within a short time frame, the amount of resources consumed by the server increases exponentially. Due to this, a delayed response to the network boot requests is likely to occur. Therefore, the server performs request optimization and inter-image deduplication to dramatically decrease the amount of responses and the total transmission volume of system images. In addition, the server flexibly switches between the response modes—unicast or broadcast—to use server resources as efficiently as possible while minimizing the time required for IoT devices to receive a response.

**Figure 8:**
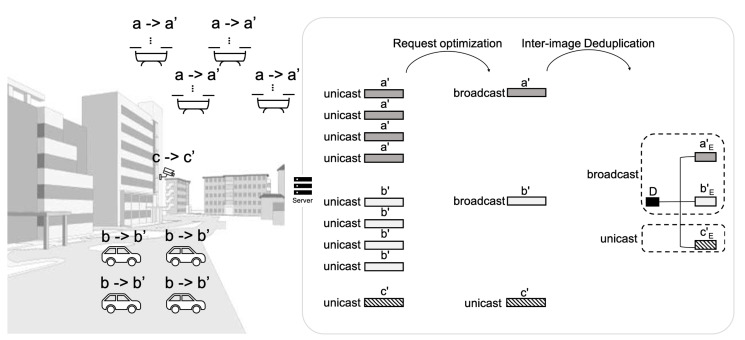
Example of deduplication of system images.

Figure 8 shows two types of deduplication which can be performed in the proposed FLEX-IoT platform. In FLEX-IoT platform, many IoT devices would use the same system images since there would be lots of IoT devices deployed for the same or very similar purpose. For example, let us say that there are four drones which need to change their system image from *a* to $a^{\prime }$, four cars which need to changes their system image from *b* to $b^{\prime }$, and a surveillance camera that needs to change its system image from *c* to $c^{\prime }$ at the same time.

**Figure 9:**
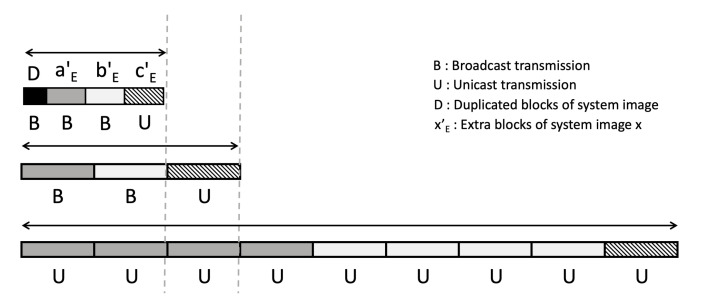
Comparison of the amount of data transmitted in three different cases.

There are three ways by which the server can send these system images. Firstly, the server can transmit system images one by one via unicast, which can be regarded as a waste of resources and time since there are a lot of duplicate system images to be transmitted. Secondly, the server can remove redundancies among the system image requests before it responds. There are four $a^{\prime }$s, four $b^{\prime }$s, and a $c^{\prime }$ to be sent in the example. If the server uses broadcast for $a^{\prime }$ and $b^{\prime }$, and uses unicast for $c^{\prime }$, it will save much time and resources to transmit all system images to many IoT devices. We call this process ’request optimization’. Third, there can still be redundancies among $a^{\prime }$, $b^{\prime }$, $c^{\prime }$. By using the deduplication table which will be explained below, we can further remove redundancies among system images to save time and resources needed to transmit those even more. We call this process ’inter-image deduplication’. We can fulfill the needs of all IoT devices with much less time and resources by using broadcast to transmit system image blocks of *D* (duplicated part of system images), $a_{E}^{\prime }$, $b_{E}^{\prime }$ (the extra blocks of $a^{\prime }$ and $b^{\prime }$) and by using unicast to transmit $c_{E}^{\prime }$ as shown in Figure 9. The extra blocks are blocks that do not duplicate one system image with another.

The three cases explained above can be generalized in the following expressions. By comparing the three expressions and choosing the method resulting in the minimum value, the server determines which method of transmission to employ when it transmits system images to the IoT devices.

$$\sum_{l=1}^{L}TR_{U}\left(DR_{l}\right)+\sum_{m=1}^{M}TR_{U}\left(R_{m}\right)$$

$$\sum_{p=1}^{P}TR_{B}\left(SR_{p}\right)+\sum_{m=1}^{M}TR_{U}\left(R_{m}\right)+\alpha \times V_{B}$$

$$TR_{B}\left(D\right)+\sum_{p=1}^{P}TR_{B}\left(DE_{p}\right)+\sum_{m=1}^{M}TR_{U}\left(RE_{m}\right)+\alpha \times V_{B}$$

The first expression represents the total transmission volume of the case where all system images are transmitted by using unicast. When there are heavily duplicated requests for the same system image ($DR_{l}$, where *l* = 1, 2, …) and requests that are not heavily duplicated ($R_{m}$, where *m* = 1, 2, …), the server can calculate the total volume of the first case by simply adding all volumes to be transferred by unicast ($TR_{U}$).

The second expression represents the case where the server uses both broadcast and unicast. When there are the heavily duplicated requests for the same system image, the server uses broadcast. However, broadcasts are resource intensive; we thus need to wisely use this method to avoid any possible side effects. A highly congested network traffic environment produces many collisions within the network, which is referred to as broadcast storm problem [26].

When the requests for the system images are not duplicated heavily, the server uses unicast. Assuming that $SR_{P}$ are the requests that duplication is removed, the volume transmitted by using broadcast ($TR_{B}$) can be written as $TR_{B}\left(SR_{p}\right)$. Again, we can write the volume of the system image that is not heavily duplicated and transmitted by using unicast as $TR_{U}\left(R_{m}\right)$. Since using broadcast can have a negative effect on the network such as broadcast storm, the proposed technique also considers the amount of broadcast transmission when deciding which method of transmission to use. We call α the broadcast overhead constant. We add the multiplication of α and the volume of system image blocks transmitted through broadcast ($V_{B}$) to the total volume of system images to be transmitted.

The third expression represents the case where the server further eliminates redundancies among different system images in addition to processing the second case. *D* is the overlapping part of the system images that all system images share. $DE_{P}$ (P=1,2,...) are the extra blocks of the system images requested by more than a certain number in the same time slice. The extra blocks are blocks that do not duplicate one system image with another. $RE_{m}$ are the extra blocks of the system images requested by less than a certain number in the same time slice. We also factor in $\alpha \times V_{B}$.

**Figure 10:**
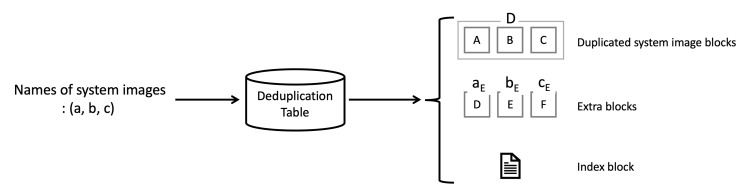
Input and output of the deduplication table.

Inter-image deduplication is performed by using the pre-calculated deduplication table as shown in Figure 10. The deduplication table accepts a combination of system images as input and it outputs deduplication results. The deduplication results consist of three types of data as shown in Figure 10. Duplicated system image blocks, which is denoted as *D*, are blocks that correspond to overlapping parts of the system images. Extra blocks, which are denoted as [name of the system image]${}_{E}$, are blocks that do not duplicate one system image with another. The index block is used to recover the original system image based on the system image blocks. Since the deduplication table is calculated and stored in advance, it does not consume significant resources on the server to get the deduplicated system images of any combination from the deduplication table. Compared to the resources required when the server transmits all system images to a lot of the IoT devices through unicast, the resources needed for acquiring deduplicated system images is minimal.

In our previous work [15], we proposed a method and scheduling technique for transmitting deduplicated system images. In this paper, we experiment with the minimum-extra-first (MEF) scheduling method among the scheduling methods proposed in the previous work. The description of the proposed MEF is as follows. Block scheduling determines the order in which the system image blocks are sent. The goal of this scheduling is to minimize the average time spent restoring all system images by scheduling the deployment order of system image blocks optimally. As this method needs to differentiate whether a block is a deduplicated part of the block or not, the MEF-scheduling should be done each time the new system image is added. This scheduling method places the system image with the smallest number of extra blocks first. Extra blocks stand for blocks that are not duplicated in a system image. Fewer extra blocks for certain system images indicate that those system images have significant redundancy. In MEF scheduling, during a series of system image deployments, previously deployed system images contain most of the system image blocks of a new system image. Therefore, the system image that is sent first with MEF scheduling can be larger than the system image sent first without scheduling, but the number of system image blocks required for deploying subsequent system images is smaller.

#### 3.3.2. Incorporation into the Existing Platforms

In this section, we describe how the proposed methods can be incorporated in existing IoT platforms. Throughout Section 3 (System Design of FLEX-IoT Platform), we proposed three main methods. They include: (1) TFTP-compatible file access control, (2) TFTP-compatible attacker deception, and (3) adaptive file transfer method. The oneM2M project is a global standards initiative for machine-to-machine communications and the internet of things. Its purpose is to standardize a common M2M and IoT service layer platform. The oneM2M functional architecture comprises the following three functions: application entity (AE), common services entity (CSE), and network service entity (NSE). The AE is an entity in the application layer that implements an M2M application service logic. A common service entity (CSE) represents an instantiation of a set of common service functions of the oneM2M service layer. For example, CSE offers service functions such as data storage and sharing with access control and authorization, event detection and notification, scheduling of data exchanges, device management, and location services. As the proposed TFTP-compatible file access control and attacker deception technique function as authorization and access control based on hash chain, these methods can be incorporated in the CSE function of oneM2M functional architecture. NSE provides services from the underlying network to the CSEs, and it supports location services, device triggering, and certain sleep modes such as PSM and long sleep cycles. Adaptive file transfer sends reboot signals to IoT devices according to the IoT platform operation schedule. Since one of the features NSE supports is device triggering, our adaptive file transfer method can make perfect use of this layer.

The Open Platform Communication Unified Architecture (OPC UA) [27] is also one of the major standardized platforms. OPC UA provides the necessary infrastructure for interoperability across the enterprise, from machine-to-machine, machine-to-enterprise, and everything in-between. OPC UA has a multi-layered architecture. This allows the incorporation of new protocols, security algorithms, encoding standards, or application-services into OPC UA. OPC UA security consists of multiple layers including application layer and communication layer. The application layer performs user authorization and authentication. On the other hand, the communication layer deals with confidentiality, integrity and app authentication. As the proposed TFTP-compatible file access control and TFTP-compatible attacker deception take responsibility of authenticating legitimate IoT devices and allowing access to the system image files, this method can be compared with the application layer of OPC UA security. There is much similarity in the two platforms. Just like the proposed system utilized hash chain, which has determined legitimate sequence of the elements, the OPC UA uses sequenced packets to eliminate exposure to message replay attacks. OPC UA specified basic functionalities to support a wide range of systems. The functionalities consist of Information Model, Information Model Access, Client-Server, and PubSub. Among these functionalities, Client-Server and PubSub functionalities can be compared with the resource-efficient network boot method. In Client-Server communication of OPC UA, each notification is for a single client with guaranteed delivery. On the other hand, PubSub is optimized for many-to-many configurations. Both platforms contain the functionalities that allow one-to-one and many-to-many data transfer.

## 4. Experimental Results

### 4.1. Experiment Environment

**Figure 11:**
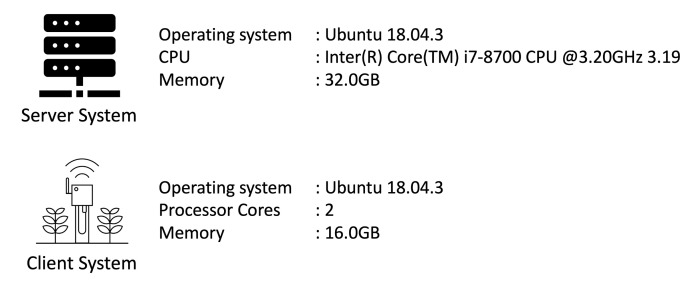
Experimental environment.

To evaluate the performance of the framework proposed in this paper, we build a virtual IoT device based on QEMU to create an IoT platform and conduct experiments. The experimental environment is shown in Figure 11.

### 4.2. Experiment on the Proposed Attacker Deception Technique

#### 4.2.1. Overhead of the Attacker Deception Technique

To implement the TFTP’s attacker deception technique, the server transmitted decoy files to the attackers whose requests have file names that do not exist on the server. We measured the time spent on the server-side to send decoy files to the attackers instead of an error message.

The time consumed to respond to the attackers when the server transmitted an error message and when the server used the attacker deception technique was measured. The average time consumed by Native TFTP that transmits an error message was 34.3698 μs. In contrast, TFTP using attacker deception technique that transmitted a decoy file consumed 66.4836 μs on average. Attack requests were transmitted 10,000 times to obtain an average value for processing time. The experimental results shows that there is only a minor overhead of an additional 32.1138 μs when the server used the proposed attacker deception. Therefore, the attacker deception technique proposed in this paper provides new security functions to the TFTP with a low overhead compared to the error handling of the conventional TFTP that transmits an error message.

The proposed TFTP-compatible file access control provides file access control capabilities to the TFTP for increased security. We simulated attacks for security evaluation as follows. The attackers’ goal was to get a system image file of the IoT devices used on the IoT platform via the TFTP. It was assumed that the attacker did not know the seed value which is used to generate the hash chain on the server and legitimate IoT devices. In the TFTP-compatible file access control, the file name on the server was set to an element of the hash chain. Since the attackers did not know the seed value and the corresponding hash chain, they generated a random 128-character string and sent an RRQ containing the string to the server in an attempt to acquire the file. In this case, the attackers would have to try as many attacks as possible to get the file. In addition, the file name of the server changes each time the IoT device sends a query message to the server. As the file name on the server is changed according to the value of the hash chain, the attackers are unable to access the file through TFTP even if they have unlimited time. In contrast, since a legitimate IoT device owns the hash chain, which is generated by using seed value shared between the server and the IoT device, it knows which value to use. Therefore, legitimate users can directly access the necessary files and download system images via the TFTP. Furthermore, based on the nature of the hash chain, IoT devices do not reuse the same value of the hash chain. Hence, it is difficult to infer the next use of the correct value based on a previous value. As explained above, with the proposed TFTP-compatible file access control, attackers will have difficulty gaining access to files on the server via the TFTP.

#### 4.2.2. Time Required to Perform Brute-Force Attack against Attacker Deception Technique

In this experiment, we evaluated the performance of the proposed attacker deception technique. The proposed attacker deception technique sends a complete 512-byte deception message instead of an error code and intentionally prolongs the response time when attackers attempt to access a file through the TFTP.

The proposed attacker deception technique uses an attacker deception message which has a larger size than the error code. Hence, the proposed deception technique adds approximately 32.1138 μs of overhead to the server for error control. However, because of this minimal overhead of approximately 32.1138 μs experienced by the server, the attackers who receive the attacker deception message have to wait for the delay time set by the TFTP. In addition, the attackers cannot decide if they need to proceed with further attacks immediately because of the decoy files that appear to be genuine files.

**Figure 12:**
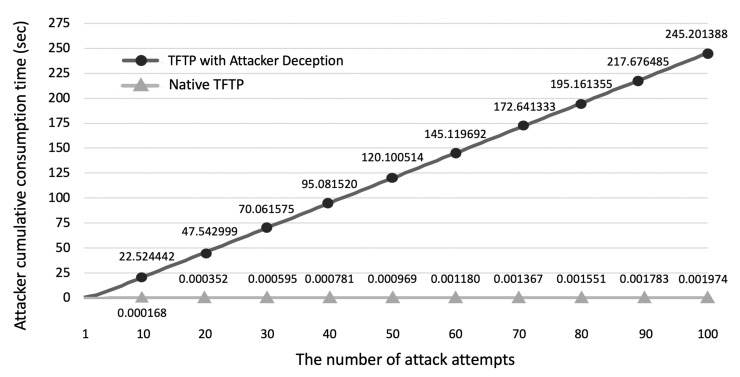
Attacker cumulative time consumption: native TFTP vs. TFTP with attacker deception.

Figure 12 presents the results of the experiment measuring the cumulative time spent by an attacker when the server sends an error code and when the server sends an attacker deception message. We used the scenario in which an attacker successfully accesses a file after 100 attempts. The figure shows the cumulative time spent by an attacker in two cases. In the first case, the server used native TFTP which responds to the attacker’s request with an error message. In the second case, the server used the attacker deception technique that sends the deception message that appears to contain a normal file instead of an error message and deliberately responds after a delay. As the attackers repeat 100 attacks, the time spent per attack increases. This means that the cumulative attack time increases exponentially. A characteristic of brute-force attacks is the rapid repetition of multiple attacks. According to the experimental results, when attackers perform a brute-force attack, fewer attacks are performed during the same period when they receive a deception message compared to when they receive an error code.

The attacker deception technique increases the amount of time that the attackers have to spend when they attempt to attack the target. To assess the security of the attacker deception technique, an attack scenario was set up as follows. The attackers’ goal was to acquire a system image of the IoT devices used on the IoT platform via the TFTP. It was assumed that they did not know the hash chain which is a series of secret values shared between the server and the legitimate IoT devices. The attackers created a random 128 character string and sent an RRQ containing the string to the server in an attempt to acquire the file. According to the TFTP-compatible file access control, only when the hash value of the SHA512 hash generated by the attacker exists on the server, the attackers can succeed in acquiring the file. In other words, the attackers can execute a successful attack only in the worst-case scenario. The TFTP without the attacker deception technique returned an error code if the file did not exist on the server. Hence, the attackers could easily determine the existence of a file based on the error code, making it easy to repeat their attack. However, because the TFTP with the attacker deception technique sends an attacker deception message that appears to contain a normal file instead of an error code, the attackers must spend time to determine if the file is a deception file. The proposed attacker deception technique increases the time spent when attackers try to execute multiple attacks. The attacker deception technique introduces additional time for invalid file access requests. All invalid file access requests are regarded as attacks in the TFTP-compatible file access control design. Therefore, the attacker deception technique does not affect the network boot of legitimate IoT devices because legitimate IoT devices with the genuine hash chain, which is a series of secret values shared with the server, do not make bad file access requests.

### 4.3. Resource-Efficient Network Boot Experiment

#### 4.3.1. Total Volume of Transferred System Image with/without Adaptive Transfer Technique

In this experiment, we evaluated the performance of an adaptive transfer scheme that monitors system image requests and dynamically changes the file transfer method. The experimental environment for adaptive transfer is listed in Table 1.

**Figure 13:**
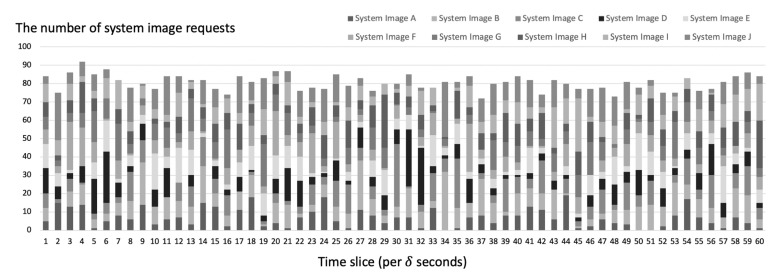
System image requests per time slice (δ seconds) simulated on the server.

Figure 13 shows the system image request scenario of a FLEX-IoT platform. This scenario was generated based on the assumption that there are 2000 IoT devices on the IoT platform. We used Poisson distribution with parameter λ=4 to generate the random time interval between requests. Each time the system image request was generated, the corresponding system image was selected based on the randomly generated probabilities which were changed per time slice. We changed these probabilities regularly on the assumption that the number of requests for each system images would dynamically change depending on the environment of the IoT platform. In addition, we assumed that the broadcast overhead constant α is 20.

**Figure 14:**
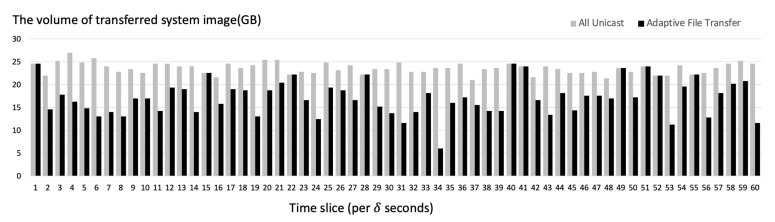
System image requests per time slice (δ seconds) simulated on the server.

Figure 14 shows the total volume of the transmitted system images when α=20. When the server used adaptive transfer that employs both unicast and broadcast to transmit system image files, the total volume of transmission per time slice was generally much lower than when the server used only unicast. When there are more than 15 duplicated requests for the same system image every δ seconds, the server adopted broadcast as the transfer method. In most cases, when there are less than 15 duplicated requests for the same system image per δ seconds, the server used unicast.

**Figure 15:**
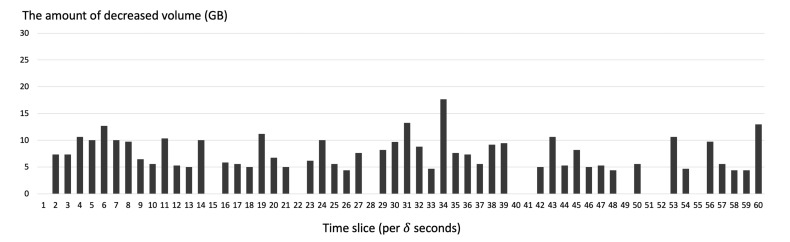
The amount of decreased volume of transferred system images per time slice (δ seconds) simulated on the server.

Figure 15 shows the amount of decreased volume of transferred system images by using adaptive file transfer. In most of the cases of our simulated result, we could see the decrease in the volume of transferred system images through adaptive file transfer.

**Figure 16:**
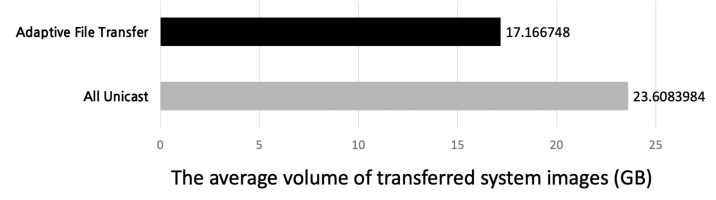
The average volume of transferred system images on the server (**Top**) using the adaptive file transfer or (**Bottom**) using only unicast.

By using the adaptive transfer technique, the server could dramatically reduce the total transmission volume of system image blocks by 27% on average in the simulation as shown in Figure 16.

However, there are some cases where the server uses only unicast because the proposed adaptive transfer technique takes into account the overhead caused by using broadcast. In this situation, the server used only unicast since the overhead caused by broadcast outweighed the benefit of the lowered total transmission volume.

**Figure 17:**
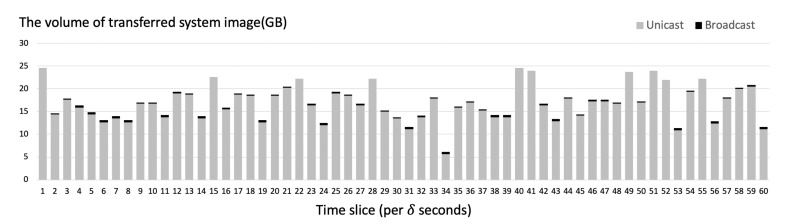
The amount of usage of broadcast and unicast per time slice (δ seconds) simulated on the server.

Figure 17 shows the amount of usage of unicast and broadcast per time slice when the broadcast overhead constant (α) is 20. As explained above, there are situations where the server uses only unicast. This is because the server factors in the overhead induced by using broadcast. We can see that the server uses broadcast only when using it is acceptable. By implementing adaptive transfer technique, the server can reduce a significant amount of data transmission volume.

#### 4.3.2. The Transfer Completion time of Block Scheduling with Data Deduplication

**Figure 18:**
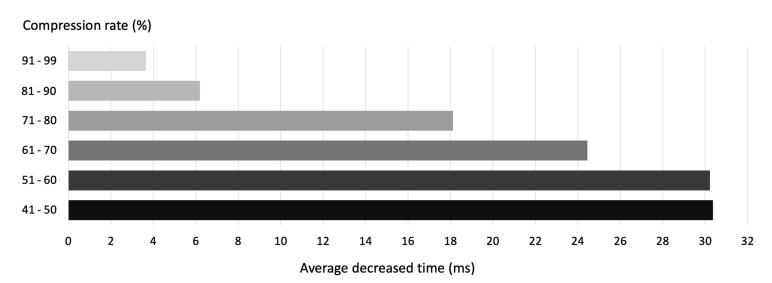
The average decreased transfer time using MEF scheduling according to compression rate.

In this experiment, we measured the file transfer completion time according to the system image block scheduling scheme. The test set used for this experiment contains 50 files consisting of 10,000 system image blocks with varying compression ratios. For this test set, we compared the average time required for transferring all 50 files with and without scheduling. When we compared the average time, the average values for each technique were calculated and compared according to the compression rate intervals. The experimental results are listed in Table 2. As shown in Figure 18, we could see enhanced performance across the entire compression rate interval when the scheduling technique was applied. When the compression rate was low, the enhancement provided by the scheduling technique increased.

## 5. Limitations

The TFTP-compatible attack deception method proposed here intentionally delays response to wrong requests. This helps prevent intensive use of network resources in case an attacker sends multiple requests over a short period of time, as in a DDoS attack. To make the IoT platform more secure, the existing DDoS defense mechanisms [28] can be applied to FLEX-IoT platform as well in order to avoid exploitation of the attacker deception method proposed in this paper.

In addition, we used simulation to verify the design of FLEX-IoT. Additional real-life implementations with performance analysis for specific types of IoT-devices will help in adoption of the proposed methods in practical scenarios.

In addition, our security assumption is that the IoT devices and the server share a seed value for generating hash chain before the devices are deployed in the IoT platform. This would require substantial management efforts. To improve the management issues, the IoT platform manager can incorporate other methods such as Diffie–Hellman key exchange [29] to share the seed value.

## 6. Conclusions

As the IoT industry continues to expand, new types of IoT platforms using network boot are being defined and implemented. However, only a few applicable control systems have been proposed for platforms using network boot. IoT devices that use network boot have the potential to reduce initial deployment and maintenance costs. Additionally, there are economic advantages to constructing IoT platforms.

Network boot has a strong dependency on FTPs. The TFTP, which is commonly used for network boot, has the advantages of minimal memory usage and simplicity while maintaining sufficient functionality. The lack of internal security of the conventional TFTP has not been a significant issue as it is typically used in environments where network security is guaranteed. However, if the TFTP is used on an IoT platform, it is impossible to guarantee the security of the network. This urges us to devise additional security measures for the TFTP and IoT platforms. In this study, we implemented TFTP-compatible file access control while complying with the design of the existing TFTP. The proposed system incurs minuscule additional overhead compared to the conventional TFTP. If an attacker wishes to acquire a file on the server through a brute-force attack, the proposed attacker deception technique increases the time required for each attack.

Additionally, IoT platforms are composed of numerous IoT devices and a network boot is likely to occur frequently. In such environments, conventional unicast file request and transmission causes excessive resource consumption on the server and leads to delayed responses. To address this issue, we reduced server resource consumption by implementing adaptive transfer based on request optimization and inter-image deduplication. The proposed system strengthens the security and the efficiency of network boot in IoT platforms. The proposed system is expected to be useful for IoT platform construction in terms of activating the market and generalizing the technology. As our system is designed to be compatible with the existing network boot environments, it can be immediately applied to real environments and used without changing protocols. We anticipate constructing an IoT platform with network boot using the system proposed in this paper to maximize infrastructure efficiency.

## Figures and Tables

**Table 1:** Adaptive transfer experimental environment.

Environment	Value
The number of IoT devices	2000
System Image Size	300 MB
Total System Image Count	10
Broadcast overhead constant (α)	20
Duplicated System Image Size	75 MB (25%)

**Table 2:** Transfer time according to compression rate intervals (ms): No scheduling vs. minimum-extra-first (MEF) scheduling.

Compression Rate	None Scheduling	MEF Scheduling
91–99	2143.683	2140.038
81–90	6036.156	6029.956
71–80	9507.26	9489.135
61–70	12,329.45	12,305
51–60	14,709	14,678.78
41–50	17,029.78	16,999.42

## References

[1] Hung M. (2017). Leading the IoT. Technical Report, Gartner. https://www.gartner.com/en/documents/3664326/iot-s-challenges-and-opportunities-in-2017-a-gartner-tre.

[2] Statista Research Department Internet of Things(IoT) Connected Devices Installed as Worldwide from 2015 to 2025. https://www.statista.com/statistics/471264/iot-number-of-connected-devices-worldwide/.

[3] Mahmoud R., Yousuf T., Aloul F., Zualkernan I. Internet of things (IoT) security: Current status, challenges and prospective measures. Proceedings of the 2015 10th International Conference for Internet Technology and Secured Transactions (ICITST).

[4] Patti E., Acquaviva A. IoT platform for Smart Cities: Requirements and implementation case studies. Proceedings of the 2016 IEEE 2nd International Forum on Research and Technologies for Society and Industry Leveraging a Better Tomorrow (RTSI).

[5] Fortino G., Savaglio C., Palau C.E., de Puga J.S., Ganzha M., Paprzycki M., Montesinos M., Liotta A., Llop M. (2018). Towards multilayer interoperability of heterogeneous IoT platforms: The INTER-IoT approach. Integration, Interconnection, and Interoperability of IoT Systems.

[6] Verma R.K., Pattanaik K., Bharti S., Saxena D. (2019). In-network context inference in IoT sensory environment for efficient network resource utilization. J. Netw. Comput. Appl..

[7] Ogawa K., Sekine H., Kanai K., Nakamura K., Kanemitsu H., Katto J., Nakazato H. Performance evaluations of iot device virtualization for efficient resource utilization. Proceedings of the 2019 Global IoT Summit (GIoTS).

[8] Hassija V., Chamola V., Saxena V., Jain D., Goyal P., Sikdar B. (2019). A survey on IoT security: Application areas, security threats, and solution architectures. IEEE Access.

[9] Zhang Z.K., Cho M.C.Y., Wang C.W., Hsu C.W., Chen C.K., Shieh S. IoT security: Ongoing challenges and research opportunities. Proceedings of the 2014 IEEE 7th International Conference on Service-Oriented Computing and Applications.

[10] O’Neill M. (2016). Insecurity by design: Today’s IoT device security problem. Engineering.

[11] Sollins K. (1992). The TFTP Protocol (Revision 2).

[12] Lear E. (2003). Uniform Resource Identifier (URI) Scheme and Applicability Statement for the Trivial File Transfer Protocol (TFTP). Technical Report, RFC 3617. https://tools.ietf.org/html/rfc3617.

[13] Isa M.A.M., Mohamed N.N., Hashim H., Adnan S.F.S., Mahmod R. A lightweight and secure TFTP protocol for smart environment. Proceedings of the 2012 International Symposium on Computer Applications and Industrial Electronics (ISCAIE).

[14] Horvat G., Zagar D., Martinovic G. (2013). STFTP: Secure TFTP protocol for embedded multi-agent systems communication. Adv. Electr. Comput. Eng..

[15] Park K.H., Park K.W. (2019). RE-NetBoot: Resource-Efficient Network Boot for IoT Platform. Res. Briefs Inf. Commun. Technol. Evol. (ReBICTE).

[16] Isa M.A.M., Hashim H., Adnan S.F.S., Manan J.l.A., Mahmod R. (2014). A secure TFTP protocol with security proofs. arXiv.

[17] Malkin G., Harkin A. RFC2347: TFTP Option Extension. https://tools.ietf.org/html/rfc2347.

[18] Mohamed N., Yussoff Y., Isa M., Hashim H. (2017). Symmetric encryption using pre-shared public parameters for a secure TFTP protocol. J. Eng. Sci. Technol..

[19] Mohamed N.N., Yussoff Y.M., Isa M.A.M., Hashim H. (2019). Extending hybrid approach to secure Trivial File Transfer Protocol in M2M communication: A comparative analysis. Telecommun. Syst..

[20] Shu Z., Yan G. Ensuring deception consistency for ftp services hardened against advanced persistent threats. Proceedings of the 5th ACM Workshop on Moving Target Defense.

[21] Takada S., Sato A., Shinjo Y., Nakai H., Sugiki A., Itano K. A p2p approach to scalable network-booting. Proceedings of the 2012 Third International Conference on Networking and Computing.

[22] Hong B., Plantenberg D., Long D.D., Sivan-Zimet M. (2004). Duplicate Data Elimination in a SAN File System. MSST. https://dblp.org/rec/conf/mss/HongPLS04.

[23] Min J., Yoon D., Won Y. (2010). Efficient deduplication techniques for modern backup operation. IEEE Trans. Comput..

[24] Muthitacharoen A., Chen B., Mazieres D. A low-bandwidth network file system. Proceedings of the Eighteenth ACM Symposium on Operating Systems Principles.

[25] Mandagere N., Zhou P., Smith M.A., Uttamchandani S. Demystifying data deduplication. Proceedings of the ACM/IFIP/USENIX Middleware’08 Conference Companion.

[26] Vegni A.M., Natalizio E. (2015). Forwarder smart selection protocol for limitation of broadcast storm problem. J. Netw. Comput. Appl..

[27] OPC Foundation Unified Architecture. https://opcfoundation.org/about/opc-technologies/opc-ua/.

[28] Mirkovic J., Reiher P. (2004). A taxonomy of DDoS attack and DDoS defense mechanisms. ACM Sigcomm Comput. Commun. Rev..

[29] Rescorla E. Rfc2631: Diffie-Hellman Key Agreement Method. https://dl.acm.org/doi/10.17487/RFC2631.

